# Acute Massive Hydroxychloroquine Overdose of 60 g in an Adult, Successfully Resuscitated: A Successful Salvage

**DOI:** 10.1155/crcc/4492860

**Published:** 2025-12-07

**Authors:** Archit Vora, Anselm Wong, Alastair Brown

**Affiliations:** ^1^ Department of Critical Care, Intensive Care Unit, St. Vincent’s Hospital, Melbourne, Australia, cmcvincent.or.kr; ^2^ Victorian Poisons Centre and Austin Toxicology Unit, Melbourne, Australia; ^3^ The University of Melbourne, Melbourne Medical School, Parkville, Australia, unimelb.edu.au

## Abstract

We present the case of a 29‐year‐old female who ingested a 60 g dose of hydroxychloroquine in a suicide attempt. This report details the acute clinical presentation, successful management strategies, and prolonged sequelae of severe hydroxychloroquine toxicity, including recurrent and delayed episodes of Torsades de Pointes (TdP) and profound metabolic derangements. Early recognition, intensive supportive care, and the use of targeted interventions—including activated charcoal administration, meticulous electrolyte correction, and vasoactive support—were critical to successful management. This case underscores the importance of prompt intervention in hydroxychloroquine overdoses and provides a review of the pathophysiology and therapeutic strategies informed by current literature and our clinical experience.

## 1. Introduction

Hydroxychloroquine, an antimalarial and immunomodulatory agent introduced in 1955, is widely prescribed for autoimmune conditions such as systemic lupus erythematosus and rheumatoid arthritis [1] and was controversially proposed as a treatment for COVID‐19 [2]. While therapeutic doses are generally well tolerated, acute overdose can result in severe toxicity, manifesting as cardiotoxicity, neurotoxicity, and metabolic disturbances. The toxic dose of hydroxychloroquine is estimated to be 20 mg/kg, with a lethal dose from as little as 4 g [3, 4]. Despite reports of fatal and near‐fatal hydroxychloroquine toxicity, there is no universally accepted management protocol. Hydroxychloroquine exhibits its toxicity through sodium and potassium channel blockade, leading to cardiovascular collapse, requiring vasopressor support as seen in this case. It can also cause profound potassium fluctuations, needing careful titration in supplementation. This case report describes the clinical course and management of a large hydroxychloroquine overdose, highlighting unique clinical manifestations and further guidance on toxidrome recognition and therapeutic strategies.

## 2. Case Presentation

**Figure 1:**
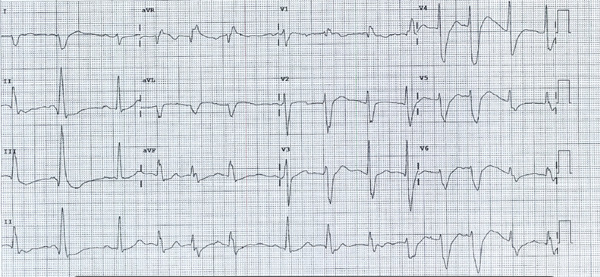
Patient′s initial ECG, 4 h post‐ingestion. The ECG shows sinus rhythm with right‐sided axis deviation. The ECG also shows polymorphic ventricular beats in couplets and triplets with a marked prolongation of the QT interval (up to 560 ms).

A 29‐year‐old female was brought to the emergency department (ED) via ambulance 3 h post‐ingestion of 60 g of hydroxychloroquine. The patient was discovered by a neighbor with altered mental status and reported self‐administering 300 tablets (with empty bottles found by the patient) of 200 mg hydroxychloroquine. Prior to arrival at the ED, the patient′s Glasgow Coma Scale (GCS) was 14 (E3V5M6). The patient weighed 59.8 kg (approximating to 1000 mg/kg massive overdose). Prehospital assessment revealed undetectable blood pressure bilaterally, with transient responsiveness to 3.5 mg of metaraminol boluses. Consequently, noradrenaline infusion was initiated with moderate hemodynamic improvement at 5 *μ*g/min, transiently improving the patient’s blood pressure to 115/83. Upon ED arrival, the patient′s vital signs were as follows: blood pressure 40–60 mmHg systolic, heart rate 80 bpm, respiratory rate 20 breaths per minute (with increased work of breathing), oxygen saturation 98% on 4 L nasal prongs, and GCS score of 14 (E4V4M6). Physical examination revealed pallor, diaphoresis, pupils equal and reactive at 3 mm, and severe nausea, with otherwise normal neurological findings. Electrocardiography (ECG) demonstrated QRS widening, QT interval prolongation, and a high ventricular ectopic burden (see Figure 1 *).*

**Figure 2:**
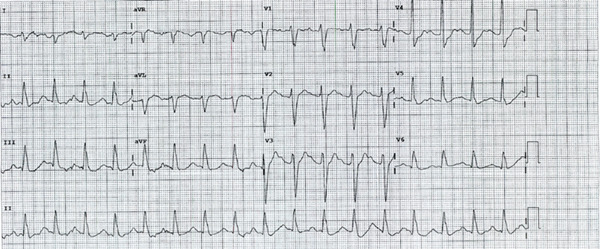
Patient′s second ECG following resuscitation, 8 h post‐ingestion. The ECG shows sinus tachycardia with right axis deviation and intraventricular conduction delay and a borderline PR interval. The ECG also shows an improvement in the QT interval (approximately 320 ms).

Following consultation with toxicology, the patient underwent rapid sequence intubation by an emergency registrar (with 40 mg of ketamine and 75 mg rocuronium) in the ED to facilitate decontamination and prevent aspiration. Post‐intubation, a 50‐g dose of activated charcoal was administered via nasogastric tube to reduce gastrointestinal absorption of the overdose. Gastric contents were aspirated and seen to be viscous in nature raising a concern of a medication suspension. Hemodynamic stabilization (heart rate 105–108, blood pressure ranging from 108/53 to 126/65 via an arterial catheter) was achieved with aggressive intravenous fluid resuscitation; although initial responses were inadequate, necessitating continued adrenaline and noradrenaline infusions (peaking at 5 and 16 *μ*g/min, respectively). The patient was subsequently transferred to the intensive care unit (ICU) for ongoing care and commenced on IV diazepam (10 mg QID) for neurogenic protection and the hypothesized reduction in the levels of chloroquine at the sodium channels at cardiac myocytes [5]. Given the initial hypokalemia (2.7 mmol/L), no sodium bicarbonate was given for the widened QRS complexes. Toxicologists provided a recommendation to consider hypertonic saline; however, given the initially stable sodium levels (132–134 mmol/L) and the subsequent improvement in QRS complexes (see Figure 2), no hypertonic saline was provided.

A critical concern was profound hypokalemia, with serum potassium reaching a nadir of 1.6 mmol/L approximately 5 h post‐ingestion. The patient′s initial potassium upon arrival to the ED was 2.7. Given the intracellular potassium shift characteristic of hydroxychloroquine toxicity rather than true depletion, potassium replacement required meticulous control to avoid rebound hyperkalemia, in consultation with toxicology. Despite careful management, the patient developed rebound hyperkalemia (7.3 mmol/L) 16 h post‐ingestion (treated with calcium chloride and an insulin/dextrose infusion), with levels fluctuating between 3 and 6 mmol/L throughout admission. Continuous arterial blood gas (ABG) monitoring guided potassium supplementation, targeting levels of 3.0–3.5 mmol/L initially, later revised to > 5.0 mmol/L following episodes of ventricular instability.

**Figure 3:**
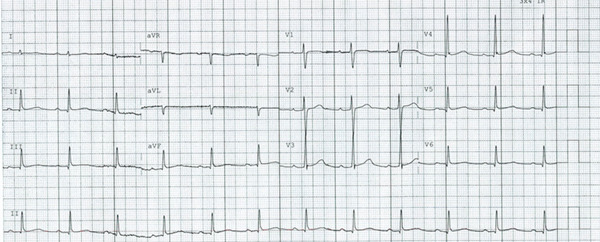
Patient′s ECG 5 days into admission. ECG shows sinus rhythm, with a right axis deviation along with a first‐degree AV block.

Within 24 h, the patient exhibited significant ventricular irritability with frequent ectopy and experienced two episodes of TdP‐related cardiac arrests, occurring 2 min apart. Both were successfully defibrillated. Considering potential proarrhythmic effects, the adrenaline infusion was discontinued. Despite aggressive magnesium and potassium supplementation, ECG monitoring continued to show prolonged QTc and the patient developed a delayed episode of TdP > 24 h post‐ingestion, which has not previously been reported in cases of hydroxychloroquine overdose. Episodes of delayed TdP are unusual in hydroxychloroquine overdoses; however, the large ingestion in this case likely contributed to continued absorption, potentially acting as a “slow release” preparation. This was managed with supplementation of magnesium (initially via 20 mmol of IV MgSO_4_ in divided doses and then 10 mmol doses when required) to achieve levels between 2.0 and 2.5 mmol/L. Isoprenaline infusion was commenced to maintain a heart rate > 80 bpm to mitigate polymorphic ventricular tachycardia risk for 48 h. The patient′s ventricular instability resolved within 4 days (see Figure 3). The magnesium targets were also subsequently eased to 1.5 mmol/L in consultation with toxicology.

**Figure 4:**
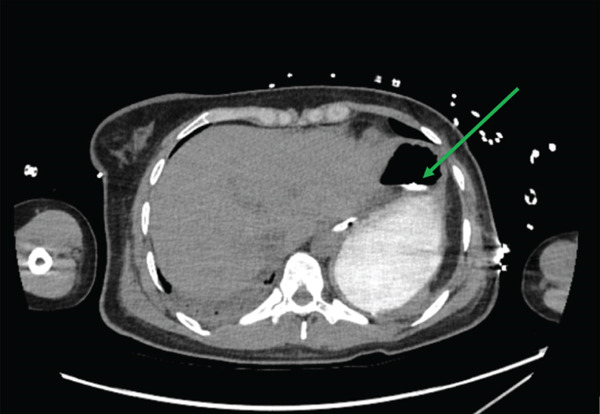
CT image of the hyperdense collection signifying a potential bezoar.

Given the massive ingestion with ongoing toxicity (multiple episodes of TdP), CT abdomen pelvis completed Day 3 into the admission (see Figure 4) revealed an 8 × 6 × 8 cm hyperdense collection within the gastric antrum, potentially suggestive of a tablet bezoar. As a result, urgent endoscopic intervention was performed by the gastroenterology team in ICU at bedside, revealing extensive activated charcoal residue but no intact tablets. Gastric contents were suctioned, and an additional dose of activated charcoal was administered per toxicology recommendation to mitigate ongoing drug absorption. The patient was extubated on Day 7 of admission and was transferred to the ward—where the patient received regular reviews from the Consultation Liaison Psychiatry team for her mental state. Subsequently, the patient was discharged from hospital on Day 9 of admission, with no long‐term damage from initial massive hydroxychloroquine overdose.

## 3. Discussion

This case highlights several key clinical challenges in the management of hydroxychloroquine overdoses in such a large quantity including prolonged cardiotoxicity through delayed absorption, metabolic toxicity, and a potential bezoar. Hydroxychloroquine is rapidly absorbed through the oral route, leading to rapid toxidrome in end organs [4]. It has a peak time to effect of approximately 3–4 h post‐ingestion [6], but toxicity can be expected in large overdoses within 1–3 ho [7]. Given the relatively rapid absorption and potentially fatal sequelae, we recommend that all hydroxychloroquine cases be discussed with a clinical toxicologist, to guide management.

Hydroxychloroquine toxicity is primarily mediated by blockade of sodium and potassium channels, leading to cardiotoxic effects, including hypotension, arrhythmias, and conduction abnormalities as witnessed in our patient. Careful consideration needs to be provided to replacing potassium in patients, as the observed hypokalemia is due to an intracellular shift of potassium rather than an overall potassium deficit [8]. As a result, patients are prone to rebound hyperkalemia, especially if treated overzealously with potassium supplementation.

We recommend early imaging of the abdomen to identify any particular “bezoars” or tablets, with a subsequent gastroscopy (if indicated) to avert ongoing toxidrome including cardiovascular toxicity. In countries with readily available CT scanners, imaging is often performed in massive drug overdoses to assess significant retention of the drug in the GI tract to determine subsequent invasive intervention, such as endoscopic removal [9].

Additionally, the occurrence of TdP greater than 24 h post‐ingestion mandates the need for prolonged cardiac monitoring, even after apparent stabilization. Intravenous magnesium sulfate, with potential repeated doses, can be administered to suppress episodes of TdP even with a normal magnesium level [10]. However, careful consideration should be given to how much magnesium a patient is provided, as toxicity such as areflexia and respiratory depression may occur, with high doses in patients who are not mechanically ventilated. Doses such as 1–2 g, such as those used in the treatment of TdP carry a low risk [11]

Dialysis is ineffective in hydroxychloroquine toxicity owing to the drug′s high volume of distribution and nondialyzable characteristics [12, 13]. In the instance of refractory cardiotoxicity, extracorporeal membrane oxygenation (ECMO) can be considered. Limited case reports describe successful treatment with ECMO despite optimal treatment [14, 15]. Given the limited experience and potential complications, ECMO should only be considered in severe cardiovascular instability, failing previously mentioned therapies. If ECMO is not readily available in severe circumstances, consideration may be given to Intralipid therapy. There is very limited evidence for intralipid therapy [5].

Life‐threatening hypoglycemia is also seen in large hydroxychloroquine overdoses, requiring extremely close monitoring of blood sugar levels, and treatment accordingly. This patient had a blood sugar level nadir of 3.2, approximately 12–13 h post‐ingestion.

The case highlighted a successful resuscitation of the patient through a multitude of treatments. In the future, potential consideration could be given to the use of higher dose benzodiazepines (diazepam). Although the patient never received any sodium bicarbonate for a widened QRS given the hypokalemia, consideration may be given in the future for potential use of hypertonic saline to aid in QRS normalization. Hydroxychloroquine levels are not readily available within standard hospital laboratories—and would not be available within a timely fashion to dictate treatment. Management should be based on the patient′s clinical picture and deterioration—as in this case.

In summary, hydroxychloroquine toxicity is a life‐threatening emergency that is challenging to manage. However, early intensive multidisciplinary care and the strategies outlined above can lead to a favorable patient outcome. Consideration of an early CT abdomen and endoscopic removal of pharmacobezoars should be made with massive ingestions of hydroxychloroquine.

## Conflicts of Interest

The authors declare no conflicts of interest.

## Funding

No funding was received for this manuscript.

## Data Availability

Data sharing is not applicable to this article as no datasets were generated or analyzed during the current study.

## References

[1] Ben-Zvi I. , Kivity S. , Langevitz P. , and Shoenfeld Y. , Hydroxychloroquine: From Malaria to Autoimmunity, Clinical Reviews in Allergy & Immunology. (2012) 42, no. 2, 145–153, 10.1007/s12016-010-8243-x, 2-s2.0-84862226725, 21221847. 21221847 PMC7091063

[2] Shukla A. M. , Archibald L. K. , Wagle Shukla A. , Mehta H. J. , and Cherabuddi K. , Chloroquine and Hydroxychloroquine in the Context of COVID-19, Drugs Context. (2020) 9, 4–5, 10.7573/dic.2020-4-5, 32373183. PMC7192209 32373183

[3] McChesney E. W. , Animal Toxicity and Pharmacokinetics of Hydroxychloroquine Sulfate, The American Journal of Medicine. (1983) 75, no. 1 supplement 1A, 11–18, 10.1016/0002-9343(83)91265-2, 2-s2.0-0020608881, 6408923. 6408923

[4] Isbister G. K. , Dawson A. , and Whyte I. M. , Electrocardiographic Manifestations of Wellens′ Syndrome, American Journal of Emergency Medicine. (2002) 20, no. 7, 638–643, 12442245. 12442245 10.1053/ajem.2002.34800

[5] Koudogbo B. , Asseko M. C. , Nguemby Mbina C. , and Laguerret-Atadou V. , Mode of Antidotal Action of Diazepam in the Treatment of Chloroquine Poisoning, Journal De Toxicologie Clinique et Experimentale. (1986) 6, no. 5, 307–312, 3795134. 3795134

[6] Florida Poison Control, Chloroquine/Hydroxychloroquine Fact Sheet. Available from: https://floridapoisoncontrol.org/wp-content/uploads/2020/04/chloroquinehydroxychloroquine-fact-sheet.pdf.

[7] ALiEM. Hydroxychloroquine, Toxicity. 2020. Available from: https://www.aliem.com/hydroxychloroquine-toxicity/.

[8] Riou B. , Barriot P. , Rimailho A. , and Baud F. J. , Treatment of Severe Chloroquine Poisoning, New England Journal of Medicine. (1988) 318, no. 1, 1–6, 10.1056/NEJM198801073180101, 2-s2.0-0023832217. 3336379

[9] Cha Y. S. , Cha S. W. , Kim S. J. , Kim Y. S. , Lee Y. , and Kim H. , The Usefulness of Non-Contrast Abdominal Computed Tomography for Detection of Residual Drugs in the Stomach of Patients With Acute Drug Overdose, Clinical Toxicology. (2019) 57, no. 7, 632–637, 10.1080/15563650.2018.1542702, 2-s2.0-85061444972. 30757921

[10] Marquardt K. and Albertson T. E. , Treatment of Hydroxychloroquine Overdose, American Journal of Emergency Medicine. (2001) 19, no. 5, 420–424, 10.1053/ajem.2001.25774, 2-s2.0-0034847933. 11555803

[11] Tzivoni D. , Banai S. , Schuger C. , Benhorin J. , Keren A. , Gottlieb S. , and Stern S. , Treatment of Torsade de Pointes With Magnesium Sulfate, Circulation. (1988) 77, no. 2, 392–397, 10.1161/01.CIR.77.2.392, 2-s2.0-0023870752. 3338130

[12] The Austin Hospital. *Chloroquine and Hydroxychloroquine Guideline* , July 2023. Available from: https://www.austin.org.au/Assets/Files/Chloroquine%2520and%2520Hydroxychloroquine%2520Guideline.%2520July%25202023.

[13] Berling I. , King J. D. , Shepherd G. , Hoffman R. S. , Alhatali B. , Lavergne V. , Roberts D. M. , Gosselin S. , Wilson G. , Nolin T. D. , and Ghannoum M. , Extracorporeal Treatment for Chloroquine, Hydroxychloroquine, and Quinine Poisoning: Systematic Review and Recommendations from the EXTRIP Workgroup, Journal of The American Society of Nephrology. (2020) 31, no. 10, 2475–2489, 10.1681/ASN.2020050564, 32963091. 32963091 PMC7609009

[14] Mongenot F. , Tessier Gonthier Y. , Derderian F. , Durand M. , and Blin D. , Traitement d’une Intoxication à l’Hydroxychloroquine Par Circulation Extracorporelle, Annales Francaises d’Anesthesie et de Reanimation. (2007) 26, no. 2, 164–167, 10.1016/j.annfar.2006.09.005, 2-s2.0-33846911498, 17092685. 17092685

[15] Rashid A. , Rehan M. , Sneck B. , and Edwards J. , ECMO Use in Massive Hydroxychloroquine Poisoning, 2019, 10.13140/RG.2.2.31417.83045.

